# Simple Diffusion as the Mechanism of Okadaic Acid Uptake by the Mussel Digestive Gland

**DOI:** 10.3390/toxins11070395

**Published:** 2019-07-06

**Authors:** Juan Blanco, Helena Martín, Carmen Mariño, Araceli E. Rossignoli

**Affiliations:** 1 Centro de Investigacións Mariñas (CIMA). Pedras de Corón, s/n. Apdo. 13, 36620 Vilanova de Arousa (Pontevedra), Spain; 2 Instituto Español de Oceanografía, Centro Costero de Vigo, Subida a Radiofaro,50, 36390 San Miguel de Oia, Spain

**Keywords:** diffusion, digestive cells, absorption, okadaic acid, transport, endocytosis, okadaic acid, uptake

## Abstract

Okadaic acid (OA) and other toxins of the diarrheic shellfish poisoning (DSP) group are accumulated and transformed mainly in many bivalves, inside the digestive gland cells. In this work the absorption of okadaic acid by those cells has been studied by supplying the toxin dissolved in water and including it in oil droplets given to primary cell cultures, and by checking if the uptake is saturable and/or energy-dependent. Okadaic acid was found to be absorbed preferentially from the dissolved phase, and the uptake from oil droplets was substantially lower. The process did not require energy and was non-saturable, indicating that it involved a simple diffusion across the cellular membrane. Some apparent saturation was found due to the quick biotransformation of OA to 7-O-acyl esters.

## 1. Introduction

**Figure 1:**
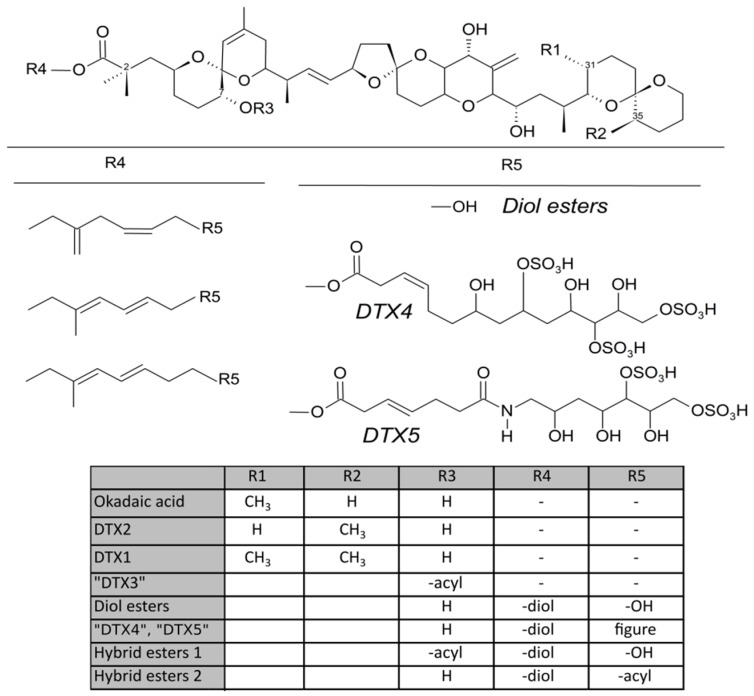
Structure of okadaic acid, dinophysistoxins-1 and 2 (DTX1 and DTX2) and some derivatives (modified from [21]).

Diarrheic shellfish poisoning is a severe gastrointestinal intoxication caused by consumption of seafood contaminated by feeding on toxigenic dinoflagellates such as some species of the genus *Dinophysis* [1,2,3,4,5,6,7,8,9] and *Prorocentrum* [10,11,12,13,14,15]. The main toxins responsible for diarrheic shellfish poisoning (DSP) are the lipophilic polyether compounds okadaic acid (OA), its isomer dinophysistoxin-2 (DTX2), its analogue dinophysistoxin-1 (DTX1) [16,17] and several fatty acid esters of these three parent toxins (generically known as DTX3) [18] (Figure 1). All these toxins are highly soluble in some organic solvents—such as methanol, acetone, chloroform or dichloromethane—but the free forms are also considerably soluble in water [19,20]. Other derivatives, as diol-esters, “DTX4” and “DTX5”, in which the carboxylic function of the OA/DTX1 esterifies other compounds, are sometimes produced by phytoplankton, but are mostly hydrolysed by the bivalves to the free toxins during digestion [21].

DSP toxins are potent inhibitors of serine/threonine protein phosphatases, especially of PP2A, but also, to a lesser extent, of PP1B and PP2B [22,23]. They have been found to be potent tumour promoters [24] and the possibility that they are also tumour inducers has been suggested [25,26]. 

The maximum allowable levels for human consumption are regulated in many countries, such as those in the EU [27]. When those levels in bivalves are exceeded, harvesting is banned, which frequently entails important economic losses for producers and distributors [9,28].

This group of toxins are accumulated almost entirely in the digestive gland of shellfish [21,29], which is the organ that absorbs most products of the extracellular digestion. Notwithstanding, the precise way by which they are absorbed by the cells of the digestive gland is not known yet, even when it could be the basis to design methods to reduce toxin accumulation and would increase the accuracy of the accumulation models. The uptake of these compounds can take place by five different types of mechanism: a) vesicular transport, b) simple diffusion, c) facilitated diffusion, d) primary active transport (pumps) and e) secondary active transport (channels and carriers). Phagocytosis, a type of vesicular transport, has been suggested by Reference [30] for highly lipophilic xenobiotics, and consists in phagocytosis of particles or lipid droplets to which lipophilic compounds with an octanol–water partition coefficient (log *P* ≥ 4) are likely associated (adsorbed or dissolved into them) [31]. Typically, the phagocyted compounds are absorbed into the cells by means of vesicles generated from the membrane (toxins do not need to pass through the membrane) and end included in lysosomes. Diffusion does not seem, a priori, a viable mechanism because the toxins are probably ionized in the digestive fluids (due to the pK_a_ of OA [32], and pH of digestive fluids [33,34]), making it difficult for them to pass through the cellular membrane. Nevertheless, the presence, in this group of toxins, of a large nonpolar portion of the molecule, and the already documented possibility of the formation of dimers with potassium that are able to pass lipid bilayers [35], make diffusion a possible mechanism of absorption. In order to identify the type of mechanism involved in OA uptake by the cells of the mussel (and by extension of other bivalves) digestive gland, we have performed a series of experiments focusing on the main differences between them. Phagocytosis can be distinguished from other mechanisms because it transports particles and not dissolved matter. Primary active transport is characterized by its use of ATP, while the remaining mechanisms do not. Secondary active transport and facilitated diffusion (which use membrane proteins) are especially efficient at low concentrations of the transported substances, but they show saturation at high concentrations because the transport capacity is limited by the number of transporter molecules in the cellular membrane. Finally, in simple diffusion, the transport is only dependent on the gradient between the outside and inside parts of the cell membrane and, consequently, at least initially, it is proportional to the concentration of the transported compound outside the cell.

In this study, we have carried out a series of experiments aimed at the identification of the uptake mechanism of OA. The first and second experiments were designed to check if phagocytosis was the transport mechanism. The third experiment focused on distinguishing between primary active transport, simple diffusion and other mechanisms (facilitated diffusion and secondary active transport).

This study has two parts. The first part (Experiments 1 and 2) aims in determining the relative importance of each uptake way in digestive gland cells of the mussel (*Mytilus galloprovincialis*). The second part (Experiment 3) aims in determining the type of transport of the dissolved OA through the cellular membrane. 

## 2. Results

### 2.1. Dissociation and Cell Culture Viability

Between 75 × 10^6^ and 91 × 10^6^ isolated cells g^−1^ of digestive gland were routinely obtained. Their initial viability was always over 78% and remained constant for at least the first 15 hours in culture. 

### 2.2. Uptake of OA Dissolved in Water and Oil

**Figure 2:**
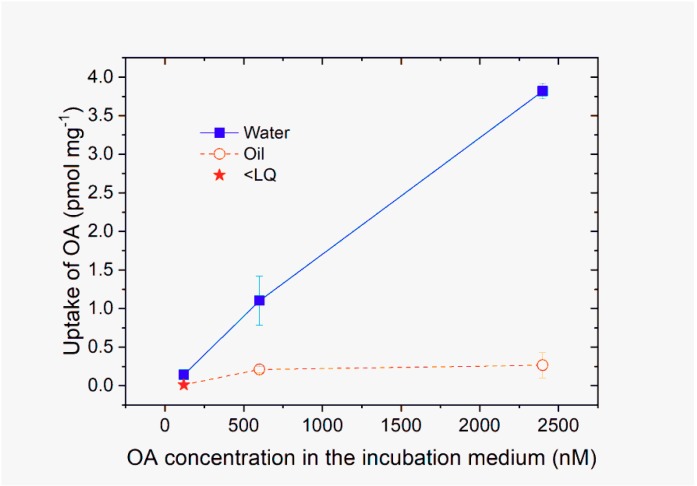
Uptake of okadaic acid (OA) by digestive gland cellular primary cultures after three hours of incubation with three OA concentrations supplied in dissolved form (water) or in oil droplets (oil). Bars indicate the standard error of the means. The star indicates that the concentration of the sample was below the limit of quantification of the LC-MS/MS method.

The uptake of OA by digestive gland tissue was higher when the toxin was supplied dissolved in water than when it was in oil droplets (Figure 2) (*p <* 0.0005). 

In the case of OA dissolved in water, the uptake by cells increased with the toxin supplied showed an almost linear relationship. However, a very slight curvature at the highest concentration seemed to exist (not statistically significant, Supplementary Materials). This trend was supported by the results of Experiment 3 in which the OA concentration was higher.

In the case of OA dissolved in oil droplets, when the concentration added to the cell culture was low (120 nM) OA in cells was below the limit of quantification (LQ). When the OA concentrations added were higher, some of the toxin uptake by cells was observed (Figure 2).

**Figure 3:**
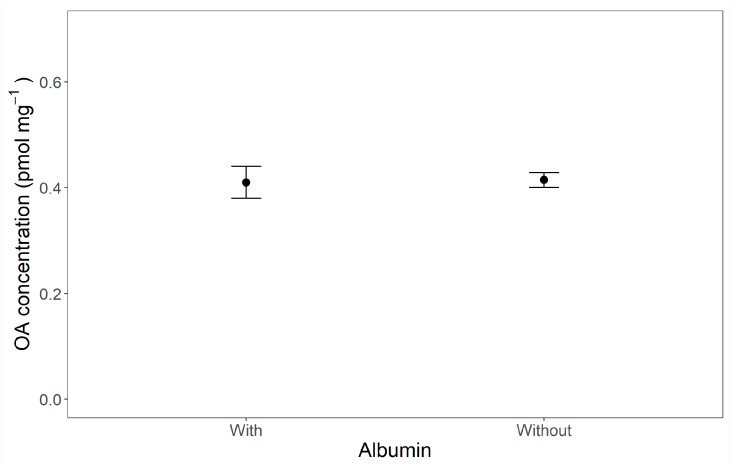
Difference in okadaic acid (OA) concentration in cellular primary cultures after incubation with OA supplied in oil droplets with and without addition of albumin to avoid aggregation of the droplets. Means (dots) ± standard errors (SE) (bars) are shown.

In the case of the toxin dissolved in oil, a very similar average uptake was found in presence or absence of an emulsifier (albumin) (0.409 ± 0.0705 and 0.414 ± 0.0197 pmol·mg^−1^, respectively; *p =* 0.909; Figure 3). The estimated values were comparable with those obtained in Experiment 1.

### 2.3. Effect of Okadaic Acid Concentration and Cyanide on OA Uptake

**Figure 4:**
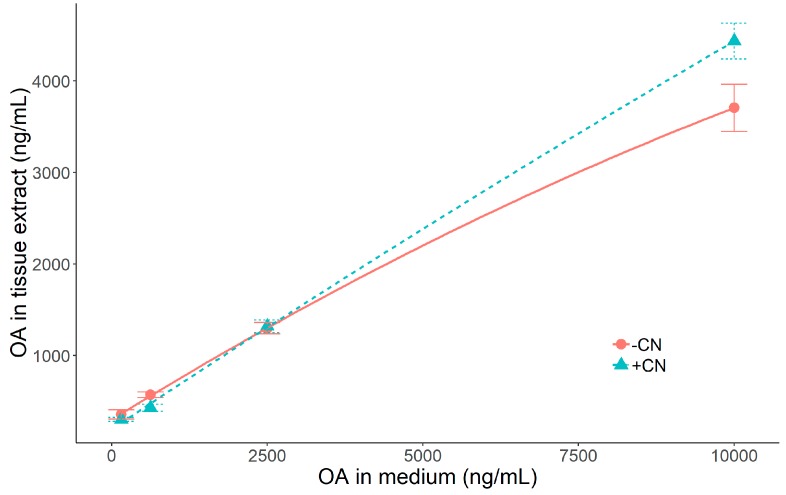
Concentration of OA in mussel digestive gland slices incubated in medium with different OA levels and with and without addition of sodium cyanide.

**Figure 5:**
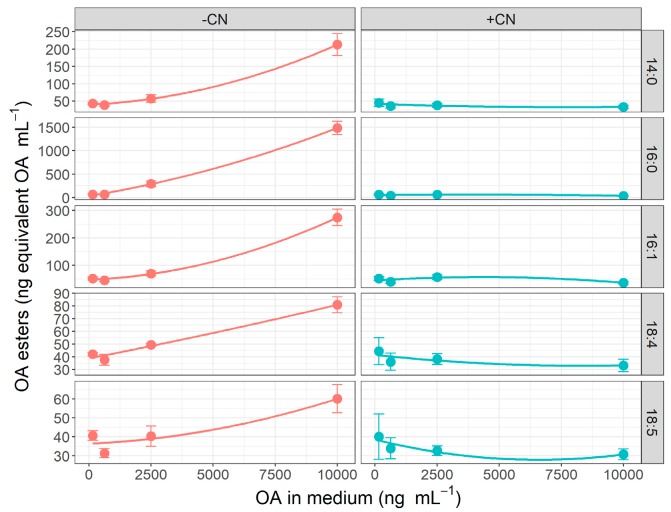
Relationship between the concentrations of the main 7-O-acyl esters of OA in slices and OA concentration in the medium, for incubations with (+CN) and without (−CN) cyanide added (mean (dots) ± standard error (bars)). The esters are designated by the number of carbon and double bonds in the chain of the fatty acid esterifying OA.

When the digestive gland slices were not exposed to sodium cyanide, the okadaic acid accumulated was not linearly related to the concentrations in the medium. However, when ATP synthesis was blocked by cyanide addition, the relationship between the concentrations of OA in medium and in slices was linear (Figure 4). Also, a detailed analysis showed a noticeable increase of concentration of 7-O-acyl esters of okadaic acid in slices not exposed to cyanide and no significant increment of those derivatives, in slices treated with cyanide (Figure 5 and Figure S1).

## 3. Discussion

According to our results, non-vesicular transport through the cell membrane, more than endocytosis, is the main way by which OA enters the digestive cells. The uptake of OA by the cells of the digestive gland was much higher from the dissolved phase than from oil droplets. This is true even when an emulsifier was added in order to avoid the aggregation of the oil droplets. The persistent low OA levels found in mussels from China have been attributed to the uptake of dissolved OA from the environment [36], which can be possible by the high stability of okadaic acid in seawater [37]. The observed subcellular distribution of OA in cells of the digestive gland of naturally contaminated mussels is also consistent with the preferential uptake of dissolved OA, because nearly 90% of the OA was in the cytosol [38] and not in lysosomes, as could be expected if the absorption would have taken place by phagocytosis or other endocytic process [30]. 

There are three main groups of transport mechanisms across cellular membranes: a) active transport, that involves membrane transporters and energy (via ATP), b) facilitated diffusion, which also involves transporters but not ATP and c) simple diffusion, in which the transported molecules pass directly across the lipidic bilayer that constitutes the cell membrane, without any consumption of energy. The uptake carried out by the mechanisms of the first two groups, is saturated at high concentrations of the transported molecules because the membrane has a limited number of transporters. They can be distinguished because the active transport requires ATP and the facilitated diffusion does not. The third mechanism is only dependent on the concentration gradient of the molecule, across the cellular membrane, thus proceeding at a rate that does not undergo saturation at high concentrations. The results obtained in Experiment 3, showed that blocking ATP synthesis with cyanide did not reduce the OA uptake, and consequently that this substance was not transported by an active mechanism. It was also clear from the results, that no saturation of the uptake at high OA concentrations took place, thus excluding facilitated diffusion and leaving simple diffusion as the main responsible mechanism.

A priori some reasons could suggest that this is not the mechanism mainly responsible for uptake. One is the high log *P* of the okadaic acid, and the other is the fact that it would be difficult to explain why the toxin is not lost at the same rate than it is taken up.

It has been suggested that the uptake of compounds with log *P* higher than 4 (as is the case of OA, [32,39]) are taken up by molluscs by endocytosis of the particles to which the compounds are adsorbed and not by diffusion [30,31]. The most likely reason why this does not happen with OA is that the hydrophilicity of OA, that is an ionisable compound, is substantially higher than that estimated by its log *P* at pH above its pKa, in fact, the coefficient of distribution log *D* (which is a function of pH and pKa; log *D* = log *P* + pKa – pH) should be used to describe the hydrophilicity instead of the partition coefficient log *P*. OA pKa has been estimated to be 4.9 [32] and ACD/Labs Software V11.02 estimates a value of 3.87 ± 0.16 from the structure. If the highest values for log *P* and pKa were assumed, log *D* at neutral pH (which is near the usual pH range in the digestive system of bivalves [33,34]) would be below four and consequently the absorption by the cells would be expected to take place from the dissolved phase, as actually happens. When intermediate values of log *P* and pKa are used to compute log *D* or when the *K_ow_* (octanol–water partition coefficients) are directly estimated [40,41], the obtained values ranged between 0.2 and 2, suggesting that OA would be taken up in dissolved and not particulate form.

In these conditions, OA being ionized could have difficulties in passing across the membrane by diffusion. Notwithstanding, it has been observed that this compound aggregates when dissolved in biological buffer [40] and that can form dimers with potassium [35] both processes “hiding” the changed portions of the molecule and making it able to pass across lipidic bilayers, as the cellular membrane. 

The asymmetry of the transport of OA, and thus, the reason why it is not lost at the same rate than incorporated, could be due to a combination of different reasons. First, when OA enters the cells it is mostly stored in high density lipoproteins (HDL) [38], the second is that, as shown in this study, OA is quickly transformed into 7-O-acyl esters (which are also stored in HDL), and third the conditions in the cytosol are different to those in the lumen of the digestive tubules.

The uptake by diffusion from the dissolved phase is supported by the fact that in mussels naturally contaminated with OA nearly 90% of the OA was in the cytosol of the digestive gland cells [38] and not in the lysosomes, as could be expected if the absorption would have taken place by phagocytosis or other endocytic processes [30]. 

Despite this, the possibility that a proportion of the OA enters the cell by the later mechanism cannot be ruled out. A study by Reference [42] using a methodology very close to that in Reference [38], found that a proportion of the OA absorbed was located in a subcellular fraction that included lysosomes. In natural conditions, it could be possible, that the proportion of OA absorbed by one or other mechanism depends on the efficiency of the extracellular step of the digestion, because OA could be included in particles from phytoplankton cells. 

Finally, in mussels in which ATP synthesis was not blocked, it has been observed a high esterification rate of OA (around 8% h^−1^ when computed from the highest OA concentration). Taking into account that OA in mussels is, in general, only partially esterified (50%–75% most usually) [19,43,44,45,46,47], this high rate suggests that the low esterification in this species is not due to a low rate but to an equilibrium in the reaction. Consequently, the proportion of esters—which regulates, at least in part, the depuration rate [47]—would not depend on time but only on the concentration of the substrates (OA and fatty acids).

## 4. Materials and Methods 

### 4.1. Biological Material and Chemicals

Mussels free or with low concentrations of toxins were obtained from culture rafts from Galicia (Spain). They were maintained overnight in tanks with aerated seawater until the start of the experiment. 

Purified water for analysis and preparation of culture medium was obtained from a MilliQ-gradient system fed with an Elix Advantage-10, both from Millipore. Acetonitrile and methanol of HPLC-grade were obtained from Rathburn (Walkerburn, Scotland, UK) and Labscan (from Galiza Analitica, Vigo, Spain), respectively. For the first experiment, okadaic acid was obtained from Calbiochem (for additions) (Merck, Madrid, Spain) and from IMB-NRC, Canada (OA-1c certificate reference material). For the second experiment, OA was obtained from Alfa-Aesar (Karlsruhe, Germany) (for additions) and from CIFGA (Lugo, Spain) (reference material). NaCl, KCl, EDTA, CaCl_2_ 2H_2_O, MgSO_4_·7H_2_O and ammonium hydroxide were supplied by Merck. HEPES (H3375-250G), gentamycin sulphate, L15 (Leibovitz) medium, albumin from bovine serum and trypan blue were obtained from Sigma-Aldrich (Madrid, Spain) and MgCl_2_·6H_2_O and sodium cyanide from Panreac (Barcelona, Spain). 

### 4.2. Experiments 1 and 2 

#### 4.2.1. Digestive Gland Dissociation 

All procedures were carried out under sterile conditions within a laminar flow hood in a thermoregulated room (18 °C ± 1°C). 

Dissociation of three digestive glands (approximately 0.5 g mussel^−1^) was carried out according to the soft dissociation procedure of Reference [48]. Mussels were dissected to obtain the digestive glands, the crystalline stylus was removed, the digestive glands placed in a beaker containing 50 mL CMFS buffer (20 mM HEPES, 500 mM NaCl, 12.5 mM KCl; pH 7.3, 1100 mOsm) supplemented with gentamycin (0.1%) and minced into small pieces (2 mm). The fragments of digestive gland were then transferred to a flask containing 250 mL CMFS buffer, including gentamycin (0.1%) and stirred gently for 2 h with a magnetic stirrer (300 rpm), taking aliquots of the cell suspension every 30 min and replacing the withdrawn suspension with fresh buffer. The obtained cell suspensions were filtered through a 100 µm sterile nylon mesh and the filtrate was centrifuged at 180*g* for 10 min. The pelleted cells were resuspended in a slightly modified L15 culture medium (0.754 g of Sigma-Aldrich L15 medium, 1.01 g of NaCl, 0.027 g of KCl, 0.03 g of CaCl_2_·2H_2_O, 0.05 g of MgSO_4_·7H_2_O, 0.195 g of MgCl_2_·6H_2_O; and 100 mL of MilliQ-gradient water, with pH 7.3 and osmotic pressure 1100 mOsm). The medium was supplemented with 0.1% gentamycin sulphate (1 mg·mL^-1^) just before use. Cells were washed with that medium by centrifugation at 180*g* for 5 min in order to remove CMFS buffer.

#### 4.2.2. Primary Cell Cultures

Primary cell cultures were carried out according to Reference [49]. The pelleted cells from digestive gland were resuspended in culture medium (L15 modified medium) and counted using a Neubauer chamber. In order to adjust the cell density to approximately 4 × 10^6^ cells·mL^−1^ the required volume of culture medium was added. The viability of the isolated cells was measured by dye exclusion using trypan blue. Finally, 100 µL aliquots of the cell suspension (approximately 4 × 10^6^ cells·mL^−1^) were distributed in a culture plate that was placed in an incubator at 18 °C. 

#### 4.2.3. Experiment 1: Uptake of Okadaic Acid Dissolved in Water and Oil

In order to test if the absorption of OA by the digestive gland cells was preferentially made from dissolved or particulate phase, 500 µL aliquots of cell culture were supplemented with either OA dissolved in water or in olive oil droplets. OA was added to a final concentration of 120, 600 and 2400 nM. The OA dissolved in oil droplets was prepared by dispersing olive oil containing OA in L15 medium (1:50, *v/v*) by sonication during 10 minutes. After the corresponding additions, cultures were incubated for 3 hours at 18 °C. All tests were carried out in duplicate.

After incubation, the cells in each well were washed three times with fresh medium (centrifugation 180*g*, 20 minutes), in order to remove the non-absorbed toxin. Finally, the medium was removed by washing with water and the absorbed toxins were extracted by adding 100% methanol (final volume 250 µL) to the pellet (previously weighed) and sonication. The obtained extract was clarified by centrifugation at 48,000*g* for 20 min. 

#### 4.2.4. Experiment 2: Effect of an Emulsifier (Albumin) on the Uptake of Okadaic Acid Dissolved in Oil

A second experiment was conducted to test if the aggregation of the oil droplets could have had an effect on the results of Experiment 1. In this experiment, the absorption of OA by cells of the digestive gland of mussels was quantified in presence or absence of albumin, which is an emulsifier [50]. A new cell culture was prepared, as in Section 4.2.1 and Section 4.2.2, and four aliquots were obtained. OA was added to each aliquot in oil droplets to a final concentration of 2340 nM. Additions to two out of the four aliquots were made with albumin as emulsifier (150 nM) and to the remaining ones without it. In preliminary experiments, with the emulsifier, no oil droplet aggregation was observed visually and by microscopic observation. Samples were incubated for 3 hours at 18 °C and OA was extracted as explained in Section 4.2.3.

### 4.3. Experiment 3: Effect of OA Concentration and Sodium Cyanide on the Uptake of the Toxin by Slices of Digestive Gland

To identify the type OA uptake mechanism, an experiment was designed to check if the uptake rate decreased at high OA concentration (characteristic of active or facilitated transport) and if blocking the ATP synthesis, by poisoning the cells with cyanide, has some effect (characteristic of active transport).

Schultz resuspension medium [51] with four levels of OA concentration (156.25, 625, 2500 and 10,000 ng·mL^−1^) was dispensed in 24-well plates (VWR, Barcelona, Spain). Ten wells were filled with each OA concentration, five of them with sodium cyanide added and the remaining five without it.

The digestive glands of the obtained mussels were sliced into 5–7 pieces of approximately 100 mg. A digestive gland slice was placed into each well, completely at random, after being weighed and washed with filtered seawater and incubated for 2 h at 20 °C (selected in a previous experiment not reported here). Additionally, one slice of each mussel was washed and directly extracted with MeOH in order to check the initial OA concentration.

After the incubation period, the slices were washed again with Milli-Q Gradient ultrapure water and extracted by sonication (Branson 450 cell disruptor) in 1 mL of MeOH. The obtained extracts were centrifuged and filtered through 0.22 µm syringe filters (Membrane Solutions, Jasco Spain, Madrid, Spain), and then analysed.

### 4.4. Toxin Analysis

Okadaic acid was analysed by LC/ESI-MS/MS using an Accela UHPLC system coupled to a triple quadrupole mass spectrometer TSQ Quantum Access MAX (Thermo Fisher Scientific, San Jose, CA, USA) equipped with a heated electrospray ionization source HESI-II. Free OA and 7-O-acyl esters were quantified by means of the analysis of the raw extracts. Chromatographic conditions were adapted from those reported by Reference [52], using a Gemini NX-C18 column (100 × 2.0 mm, 3 µm) from Phenomenex (Torrance, CA, USA) maintained at 40 °C. Mobile phases A and B were, respectively, water and MeCN/water (90/10, *v/v*), both containing 6.7 mM NH_4_OH (pH = 11). The following linear gradient at a flow rate 400 µL min^−1^ was used: start at 25%B, hold for 1 min, increase from 25%B to 95%B over 4 min, hold at 95%B for 3 min, return to initial conditions over 2 min and re-equilibrate for 2 min. The total run time was 12 minutes, including column re-equilibration. Injection volume was 5 µL.

The ion transfer tube temperature and the HESI-II vaporizer temperature were set at 360 °C and 110 °C, respectively. Nitrogen was used as sheath and auxiliary gas at 60 and 10 arbitrary gas pressure units, respectively. The mass spectrometer was operated in negative ionization mode with a spray voltage of 3500 V. Detection was carried out in the multiple reaction monitoring (MRM) mode using argon (>99.999%) as collision-induced-dissociation (CID) gas at a pressure of 1.5 mTorr. For OA, the transitions 803.5 > 255.2 and 803.5 > 563.5 *m/z,* with collision energies of 48 and 43 eV, in negative ionization mode, were used for quantification and confirmation, respectively. For the 7-O-acyl esters, the transitions from the *m/z* corresponding to their Na adduct and 705.5, with a collision energy of 52, were used (Table S1).

Quantification of OA was carried out by external calibration using certified reference solutions in methanol. Relevant matrix effects were not observed when assessed using the post-extraction addition method (spiking). The concentrations of the main 7-O-acyl esters of OA were approximately quantified (only for comparison of the treatments) as OA-equivalents, by assuming that they had same response in the mass spectrometer as the sodium adduct of OA.

### 4.5. Statistical Analysis

The statistical significance of the treatments in all experiments was checked by means of ANOVA and linear regression, with R Statistical Package. 

## Supplementary Materials

The following are available online at https://www.mdpi.com/2072-6651/11/7/395/s1, Figure S1: Chromatograms of selected 7-O-acyl esters of OA from slices incubated with 10,000 ng·mL^−1^ of OA, Table S1: Transitions for the quantification of the 7-O-acyl esters of OA.
