# Supplementary material for: Simple Diffusion as the Mechanism of Okadaic Acid Uptake by the Mussel Digestive Gland
Source: Toxins (Basel). 2019 Jul 6;11(7):395. doi: 10.3390/toxins11070395 (PMC6669496; doi:10.3390/toxins11070395)
Supplement: Supplementary file 1 [file toxins-11-00395-s001.pdf]

Supplementary Material

Simple Diffusion as the Mechanism of Okadaic Acid Uptake by the Mussel Digestive Gland

Methods

Table S1. Transitions for the quantification of the 7-O-Acyl esters of OA.

Compound	Parent m/z	Product m/z	CE	Polarity
Na-OA	827.5	723.4	55	+
Na-14:0-OA	1037.501	705.5	52	+
Na-18:4-OA	1085.501	705.5	52	+
Na-18:3-OA	1087.501	705.5	52	+
Na-16:1-OA	1063.501	705.5	52	+
Na-20:5-OA	1111.501	705.5	52	+
Na-15:0-OA	1051.501	705.5	52	+
Na-16:0-OA	1065.501	705.5	52	+
Na-17:1-OA	1077.501	705.5	52	+
Na-18:1-OA	1091.501	705.5	52	+
Na-20:4-OA	1113.501	705.5	52	+
Na-22:6-OA	1137.501	705.5	52	+
Na-18:2-OA	1089.501	705.5	52	+
Na-17:0-OA	1079.501	705.5	52	+
Na-20:2-OA	1117.501	705.5	52	+
Na-18:0-OA	1093.501	705.5	52	+
Na-20:1-OA	1119.501	705.5	52	+

Statistical analysis

All procedures were carried out with R statistical package unless specifically stated.

Experiment 1.

Differences between Water and oil dissolved OA (linear regression with a continuous variable and one categorical one (water-oil).

```
> regresOilWater<-lm(pmol.mg.OA~ConcMedium*TRATAMIENTO, data= dataOilW
ater)
> summary(regresOilWater)

Call:
lm(formula = pmol.mg.OA ~ ConcMedium * TRATAMIENTO, data = dataOilWate
r)

Residuals:
     Min       1Q   Median       3Q      Max 
-0.20876 -0.12257 -0.05755  0.08971  0.43119 

Coefficients:
                             Estimate Std. Error t value Pr(>|t|)    
(Intercept)                 6.872e-02  1.249e-01   0.550    0.597    
ConcMedium                  8.934e-05  8.732e-05   1.023    0.336    
TRATAMIENTOWATER           -3.006e-02  1.766e-01  -0.170    0.869    
ConcMedium:TRATAMIENTOWATER 1.495e-03  1.235e-04  12.107    2e-06 ***
---
Signif. codes:  0 ‘***’ 0.001 ‘**’ 0.01 ‘*’ 0.05 ‘.’ 0.1 ‘ ’ 1

Residual standard error: 0.2099 on 8 degrees of freedom
Multiple R-squared:  0.9839,	Adjusted R-squared:  0.9778 
F-statistic: 162.9 on 3 and 8 DF,  p-value: 1.647e-07
```

Relationship OA in cells – concentration of OA in medium

```
> dataWater<-dataOilWater %>% filter(TRATAMIENTO=="WATER")
> regresWaterConc<-lm(pmol.mg.OA~poly(ConcMedium,2), data = dataWater )
> summary(regresWaterConc)

Call:
lm(formula = pmol.mg.OA ~ poly(ConcMedium, 2), data = dataWater)

Residuals:
       1        2        3        4        5        6 
 0.10168  0.31998  0.03393 -0.10168 -0.31998 -0.03393 

Coefficients:
                     Estimate Std. Error t value Pr(>|t|)    
(Intercept)            1.6865     0.1125  14.993 0.000644 ***
poly(ConcMedium, 2)1   3.8090     0.2755  13.824 0.000819 ***
poly(ConcMedium, 2)2  -0.2031     0.2755  -0.737 0.514435    
---
Signif. codes:  0 ‘***’ 0.001 ‘**’ 0.01 ‘*’ 0.05 ‘.’ 0.1 ‘ ’ 1

Residual standard error: 0.2755 on 3 degrees of freedom
Multiple R-squared:  0.9846,	Adjusted R-squared:  0.9743 
F-statistic: 95.83 on 2 and 3 DF,  p-value: 0.001913
```

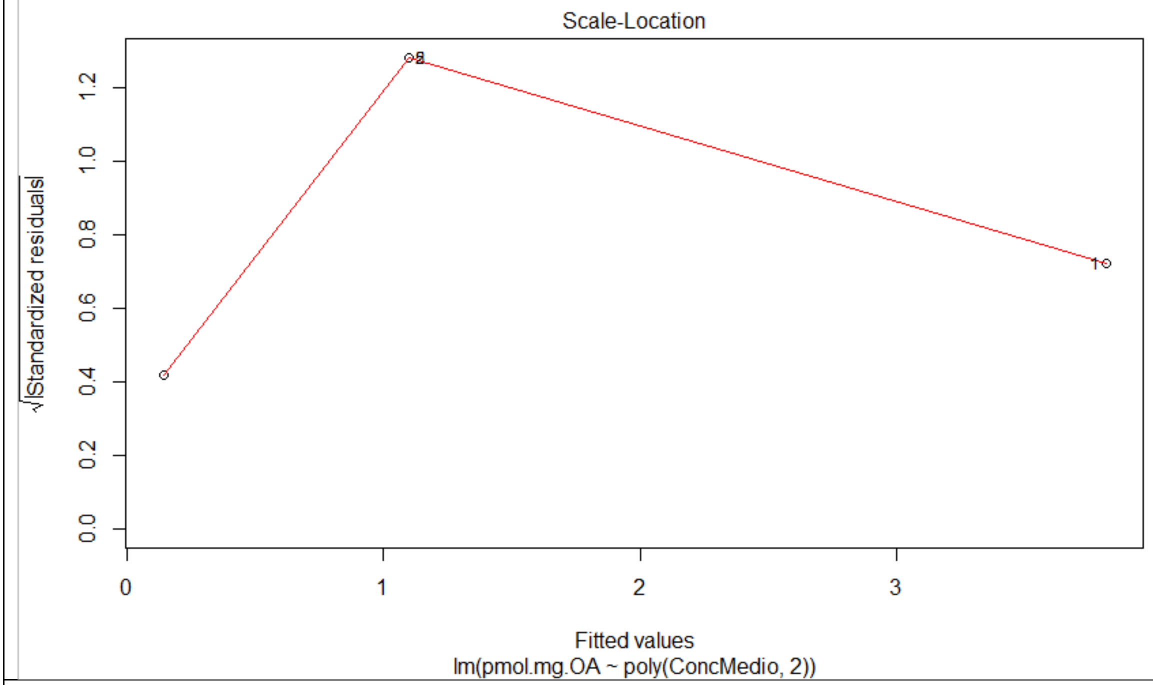

Distribution of the standardized residuals showing curvature (middle points higher than extreme ones)

Experiment 2 Analysis of the effect of albumin

Anova of the results obtained

```
> regresAlbumin<-lm(pmol.mg~Albumin,data=dataAlbumin)
> anovaAlbumin<-aov(regresAlbumin)
> summary(anovaAlbumin)
            Df    Sum Sq   Mean Sq F value Pr(>F)
Albumin      1 0.0000232 0.0000232   0.021  0.898
Residuals    2 0.0022313 0.0011156
```

Computations of the power of the used tests:

The expected difference, from the previous experiment is 3. With two replicates and using a standard power of 0.8 a difference of 1.7 could be detected with a significance level of 0.001, and a difference of 0.5 with a power of 0.999 and a significance level of 0.05.

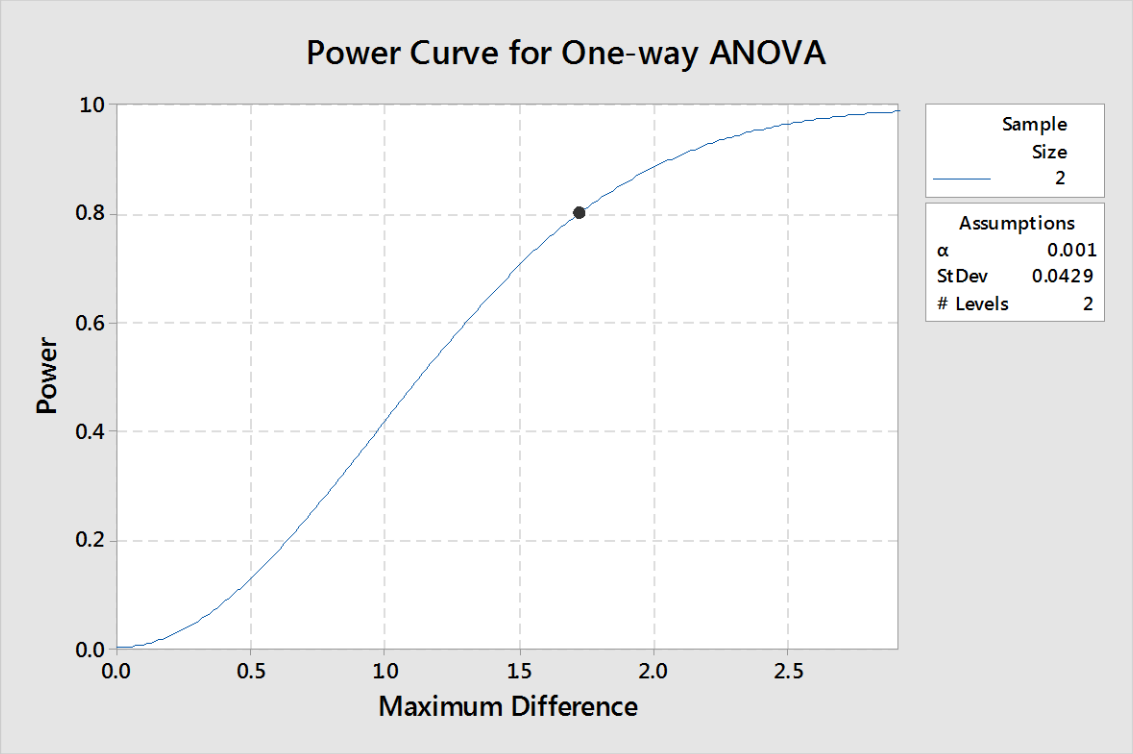

Detectable difference with a power (standard) of 0.8 and α = 0.001 (computed with Minitab 18)) using the highest variance of the treatments (very conservative estimate, because the Residual Mean Square from the ANOVA, which is much smaller, could be used).

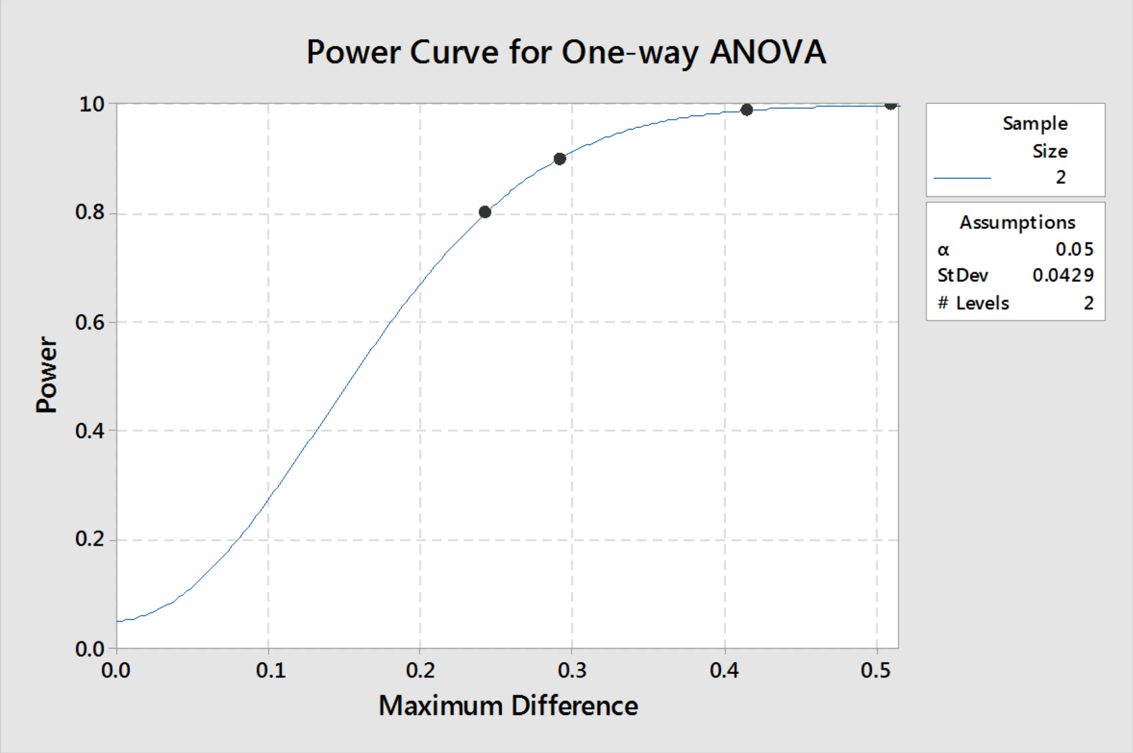

Detectable difference with power of 0.8, 0.9, 0.99 and 0.999 (increasing to the right) and α = 0.05 (computed with Minitab 18)) using the highest variance of the treatments (very conservative estimate, because the Residual Mean Square from the ANOVA, which is much smaller, could be used).

Experiment 3.

Differences between addition and not addition of CN

```
> regresPieces<-lm(Conc.ng.g~ConcMedium*cyan,data=dataPieces)
> summary(regresPieces)

Call:
lm(formula = Conc.ng.g ~ ConcMedium * cyan, data = dataPieces)

Residuals:
    Min      1Q  Median      3Q     Max 
-908.37 -136.45  -31.04   93.94 1625.02 

Coefficients:
                    Estimate Std. Error t value Pr(>|t|)    
(Intercept)        365.30615   78.04278   4.681 1.22e-05 ***
ConcMedium           0.33617    0.01511  22.244  < 2e-16 ***
cyan+CN           -148.01872  110.36915  -1.341 0.183875    
ConcMedium:cyan+CN    0.08639    0.02137   4.042 0.000126 ***
---
Signif. codes:  0 '***' 0.001 '**' 0.01 '*' 0.05 '.' 0.1 ' ' 1

Residual standard error: 378 on 76 degrees of freedom
Multiple R-squared:  0.9439,	Adjusted R-squared:  0.9417 
F-statistic: 426.4 on 3 and 76 DF,  p-value: < 2.2e-16
```

Relationship OA in digestive gland pieces and OA in the culture medium

It can be observed that the importance (coefficient and Pr) of the quadratic component of the regression is higher in the pieces without cyanide added. In any case, the deviation from the linearity is not significant.

With Cyanide added

```
> regresPiecesconCN<-lm(Conc.ng.g~poly(ConcMedium,2),data=dataPiecesco
nCN)
> summary(regresPiecesconCN)

Call:
lm(formula = Conc.ng.g ~ poly(ConcMedium, 2), data = dataPiecesconCN)

Residuals:
    Min      1Q  Median      3Q     Max 
-849.36 -122.20  -20.32   89.54  819.08 

Coefficients:
                     Estimate Std. Error t value Pr(>|t|)    
(Intercept)           1620.31      52.56  30.826   <2e-16 ***
poly(ConcMedium, 2) 110569.64     332.44  31.794   <2e-16 ***
poly(ConcMedium, 2)2  -120.60     332.44  -0.363    0.719    
---
Signif. codes:  0 '***' 0.001 '**' 0.01 '*' 0.05 '.' 0.1 ' ' 1

Residual standard error: 332.4 on 37 degrees of freedom
Multiple R-squared:  0.9647,	Adjusted R-squared:  0.9628 
F-statistic: 505.5 on 2 and 37 DF,  p-value: < 2.2e-16
```

Without Cyanide added

```
>
> regresPiecessinCN<-lm(Conc.ng.g~poly(ConcMedium,2),data=dataPiecessi
nCN)
> summary(regresPiecessinCN)

Call:
lm(formula = Conc.ng.g ~ poly(ConcMedium, 2), data = dataPiecessinCN)

Residuals:
    Min      1Q  Median      3Q     Max 
-886.90 -144.37  -31.88  108.48 1646.49 

Coefficients:
                      Estimate Std. Error t value Pr(>|t|)    
(Intercept)            1481.50      66.94  22.132   <2e-16 ***
poly(ConcMedium, 2)1   8408.80     423.37  19.862   <2e-16 ***
poly(ConcMedium, 2)2   -354.01     423.37  -0.836    0.408    
---
Signif. codes:  0 '***' 0.001 '**' 0.01 '*' 0.05 '.' 0.1 ' ' 1

Residual standard error: 423.4 on 37 degrees of freedom
Multiple R-squared:  0.9144,	Adjusted R-squared:  0.9098 
F-statistic: 197.6 on 2 and 37 DF,  p-value: < 2.2e-16
```

Comparison of the two highest concentration levels with and without cyanide by two way anova.

This comparison is carried out because the errors in the lowest levels could be important in determining the curvature. In this case the existence of curvature is approximated by testing if the highest levels of the treatment with cyanide are higher that the corresponding ones of those corresponding to the treatment with cyanide.

The pieces treated with cyanide acquired significant higher amounts of OA than those not treated.

```
> dataPiecesMasde2000<-dataPieces %>% filter( ConcMedium>2000)
> regresDataPiecesMasde2000<-lm(Conc.ng.g~ConcMedium+cyan,data=dataPie
cesMasde2000)
> anovaPiecesMasde2000<-aov(regresDataPiecesMasde2000)
> summary(regresDataPiecesMasde2000)

Call:
lm(formula = Conc.ng.g ~ ConcMedium + cyan, data = dataPiecesMasde2000)

Residuals:
     Min       1Q   Median       3Q      Max 
-1064.07  -342.40   -33.11   256.84  1469.32 

Coefficients:
             Estimate Std. Error t value Pr(>|t|)    
(Intercept) 198.48229  192.07888   1.033   0.3082    
ConcMedium    0.36842    0.02343  15.722   <2e-16 ***
cyan+CN     375.23478  175.75053   2.135   0.0394 *  
---
Signif. codes:  0 '***' 0.001 '**' 0.01 '*' 0.05 '.' 0.1 ' ' 1

Residual standard error: 555.8 on 37 degrees of freedom
Multiple R-squared:  0.8719,	Adjusted R-squared:  0.8649 
F-statistic: 125.9 on 2 and 37 DF,  p-value: < 2.2e-16
```

Regression Acyl esters with OA concentration in Medium . Effect of cyanide

Regression analysis showing the statistical differences in slope between the incubation with and without cyanide addition (term interaction ConcMedio-Cian)

```
> LSregresAcil<-LSdatosAcilSep %>% map(~lm(.$Conc.ng.g~.$ConcMedio*.$c
ian))
```

OA-C14:0

```
> summary(LSregresAcil[[1]])

Call:
lm(formula = .$Conc.ng.g ~ .$ConcMedio * .$cian)

Residuals:
    Min      1Q  Median      3Q     Max 
-57.674 -12.631   1.315  10.076 122.303 

Coefficients:
                       Estimate Std. Error t value Pr(>|t|)    
(Intercept)           29.220880   8.387842   3.484  0.00135 ** 
.$ConcMedio            0.018056   0.001593  11.338 2.87e-13 ***
.$cian+CN             11.689187  11.740248   0.996  0.32625    
.$ConcMedio:.$cian+CN -0.018841   0.002251  -8.370 7.16e-10 ***
---
Signif. codes:  0 ‘***’ 0.001 ‘**’ 0.01 ‘*’ 0.05 ‘.’ 0.1 ‘ ’ 1

Residual standard error: 28.14 on 35 degrees of freedom
Multiple R-squared:  0.822,	Adjusted R-squared:  0.8067 
F-statistic: 53.87 on 3 and 35 DF,  p-value: 3.355e-13
```

OA-C16:0

```
> summary(LSregresAcil[[2]])

Call:
lm(formula = .$Conc.ng.g ~ .$ConcMedio * .$cian)

Residuals:
    Min      1Q  Median      3Q     Max 
-387.32  -19.19   -3.26   38.91  447.54 

Coefficients:
                       Estimate Std. Error t value Pr(>|t|)    
(Intercept)           -8.892143  35.546387  -0.250    0.804    
.$ConcMedio            0.147897   0.006749  21.914   <2e-16 ***
.$cian+CN             67.069678  49.753369   1.348    0.186    
.$ConcMedio:.$cian+CN -0.149700   0.009539 -15.693   <2e-16 ***
---
Signif. codes:  0 ‘***’ 0.001 ‘**’ 0.01 ‘*’ 0.05 ‘.’ 0.1 ‘ ’ 1

Residual standard error: 119.2 on 35 degrees of freedom
Multiple R-squared:  0.9458,	Adjusted R-squared:  0.9412 
F-statistic: 203.6 on 3 and 35 DF,  p-value: < 2.2e-16
```

OA-C16:1

```
> summary(LSregresAcil[[3]])

Call:
lm(formula = .$Conc.ng.g ~ .$ConcMedio * .$cian)

Residuals:
    Min      1Q  Median      3Q     Max 
-74.803 -15.703  -2.202  11.748  88.071 
```

```
Coefficients:
                        Estimate Std. Error t value Pr(>|t|)    
(Intercept)            32.361821   8.497296   3.808 0.000541 ***
.$ConcMedio             0.023780   0.001613  14.740  < 2e-16 ***
.$cian+CN              17.648646  11.893448   1.484 0.146787    
.$ConcMedio:.$cian+CN -0.024997   0.002280 -10.962 7.26e-13 ***
---
Signif. codes:  0 ‘***’ 0.001 ‘**’ 0.01 ‘*’ 0.05 ‘.’ 0.1 ‘ ’ 1

Residual standard error: 28.5 on 35 degrees of freedom
Multiple R-squared:  0.8855,	Adjusted R-squared:  0.8757 
F-statistic: 90.23 on 3 and 35 DF,  p-value: < 2.2e-16
```

OA-C18:4

```
> summary(LSregresAcil[[4]])

Call:
lm(formula = .$Conc.ng.g ~ .$ConcMedio * .$cian)

Residuals:
    Min      1Q  Median      3Q     Max 
-18.628  -9.056   0.483   5.285  43.220 

Coefficients:
                        Estimate Std. Error t value Pr(>|t|)    
(Intercept)           38.3897982  3.8596464   9.946 1.34e-11 ***
.$ConcMedio            0.0042463  0.0007175   5.918 1.10e-06 ***
.$cian+CN              2.1194173  5.3441750   0.397    0.694    
.$ConcMedio:.$cian+CN -0.0050039  0.0010135  -4.937 2.07e-05 ***
---
Signif. codes:  0 ‘***’ 0.001 ‘**’ 0.01 ‘*’ 0.05 ‘.’ 0.1 ‘ ’ 1

Residual standard error: 12.66 on 34 degrees of freedom
  (1 observation deleted due to missingness)
Multiple R-squared:  0.5915,	Adjusted R-squared:  0.5555 
F-statistic: 16.41 on 3 and 34 DF,  p-value: 9.146e-07
```

OA-C18:5

```
> summary(LSregresAcil[[5]])

Call:
lm(formula = .$Conc.ng.g ~ .$ConcMedio * .$cian)

Residuals:
    Min      1Q  Median      3Q     Max 
-21.138  -8.575  -1.095   7.011  34.952 

Coefficients:
                        Estimate Std. Error t value Pr(>|t|)    
(Intercept)           34.7677030  4.4505613   7.812 2.12e-08 ***
.$ConcMedio            0.0025070  0.0007514   3.337  0.00248 ** 
.$cian+CN              1.0223001  5.9562488   0.172  0.86500    
.$ConcMedio:.$cian+CN -0.0030892  0.0011475  -2.692  0.01205 *  
---
Signif. codes:  0 ‘***’ 0.001 ‘**’ 0.01 ‘*’ 0.05 ‘.’ 0.1 ‘ ’ 1

Residual standard error: 12.28 on 27 degrees of freedom
  (8 observations deleted due to missingness)
Multiple R-squared:  0.3984,	Adjusted R-squared:  0.3315 
F-statistic:  5.96 on 3 and 27 DF,  p-value: 0.002962
```

Other information

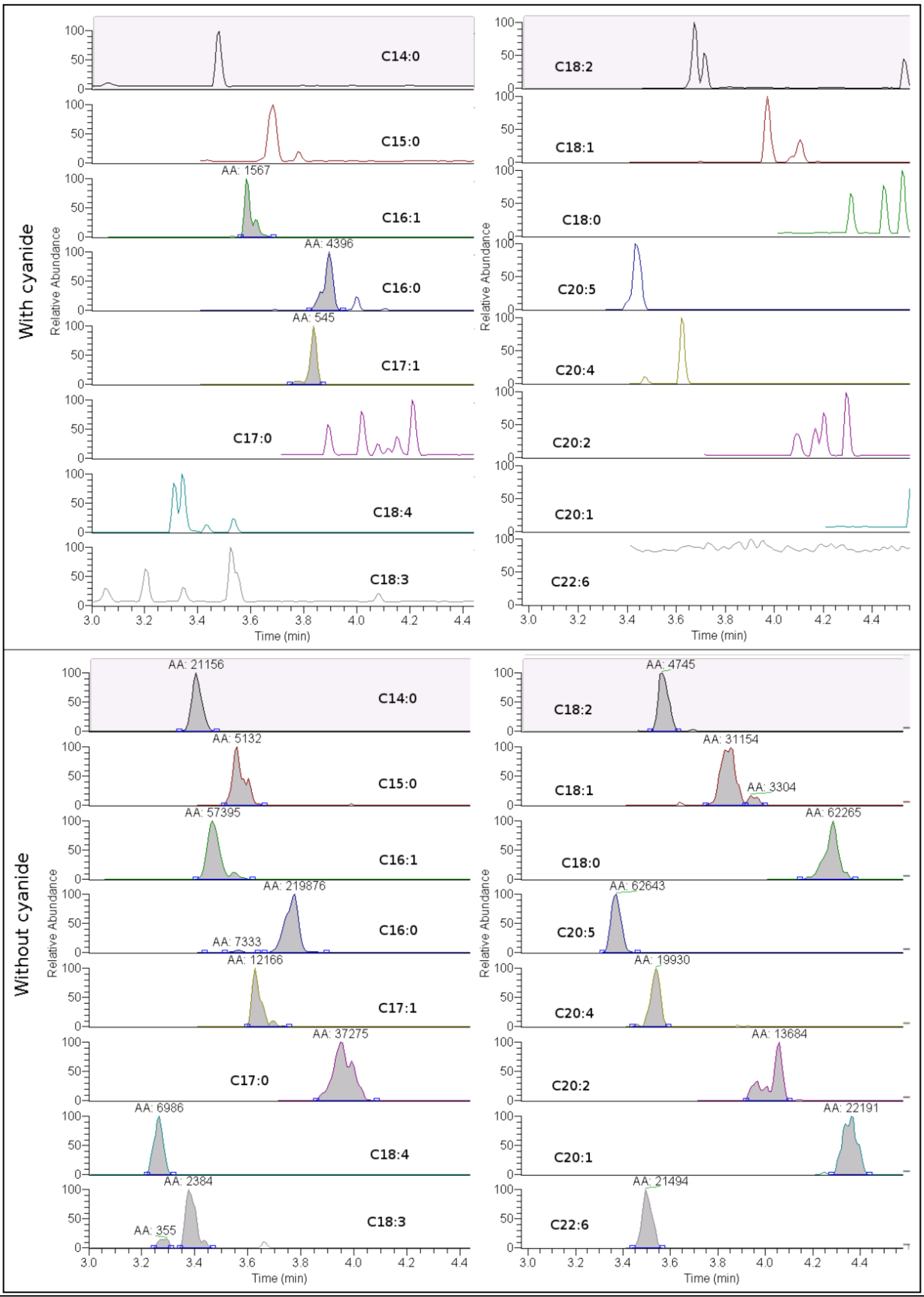

Figure S1. Chromatograms of selected 7-O-acyl esters of OA from slices incubated with 10000 ng·mL^{-1} of OA. The upper and lower panels correspond to samples incubated with and without cyanide, respectively. The areas (AA) of the peaks that were sufficiently large to be integrated are shown. Peaks below that level correspond to very low signals and are probably artifacts. The code in each chromatogram corresponds to the number of carbons and double bonds of the fatty acid which esterifies OA and consequently several isomers could be present in the same chromatogram thus generating several peaks.
